# A Quad-Band Highly Selective Frequency Selective Surface with Ultra-Wideband Rejection

**DOI:** 10.3390/mi15010126

**Published:** 2024-01-11

**Authors:** Minrui Wang, Zheng Xiang, Yi Li, Baoyi Xu, Long Yang

**Affiliations:** The State Key Laboratory of Integrated Services Networks, Xidian University, Xi’an 710071, China; mrwang614@163.com (M.W.); zhx@mail.xidian.edu.cn (Z.X.); byxu@xidian.edu.cn (B.X.); lyang@xidian.edu.cn (L.Y.)

**Keywords:** frequency selective surface (FSS), highly selective, quad-band, ultra-wideband rejection

## Abstract

In this paper, a highly selective quad-band frequency selective surface (FSS) with ultra-wideband rejection is presented. The proposed FSS structure was developed by cascading five metallic layers by three thin dielectric substrates. The five metallic layers are composed of two bent slot layers, two metallic square rings, and a metal patch. The dimensions of the unit cell are 0.13$\lambda_{0}$× 0.13$\lambda_{0}$× 0.18$\lambda_{0}$ ($\lambda_{0}$ is the free-space wavelength at the first operating frequency). The proposed structure achieves four transmission bands and has two wide stop-bands located at 1 to 5.5 GHz and 14 to 40 GHz, with a suppressed transmission coefficient below −20 dB. In order to verify the simulation results, an FSS prototype was fabricated and measured. It can be observed that the measured results are in favorable agreement with the simulation results. Its multiple narrow passbands and highly selective and ultra-wideband rejection properties ensure that our design can play a significant role in narrowband antennas, spatial filters, and many other fields.

## 1. Introduction

Frequency selective surfaces (FSSs) are two-dimensional periodic structures composed of metal arrays or aperture units on dielectric substrates, which can be regarded as spatial filters. The filtering characteristics of FSSs are reflected in the transmission or reflection specific electromagnetic waves at specific frequency bands [1]. Owing to their unique filtering properties, FSSs can be used in radomes [2,3], radar cross-section (RCS) reduction [4], antennas [5], etc. Particularly in narrowband antennas, radomes with narrow passbands have a highly selective filtering response to reduce the out-of-band RCS of the narrowband antennas and effectively suppress interference and interfering signals.

Multi-band FSSs have the advantages of saving spectrum resources and improving anti-interference ability. With the application of multi-band communication in civilian communication equipment and modern military systems, it is very necessary to design multi-band FSSs to satisfy the performance requirements of these communication systems [6,7].

In recent years, many studies on designing multi-band FSSs have been reported by introducing multilayer structures. In [8], a second-order dual-band FSS is mentioned, employing a particular topology composed of resonant and nonresonant structures. A miniaturized and low-profile FSS is presented in [9], where the convoluted design is used in the proposed FSS to achieve three passbands. By cascading two gridded triple square loop (G-TSL) structures and a double square loop (DSL) structure, a quad-band FSS is presented in [10]. However, most of the above FSS studies neglected the out-of-band performance.

As for practical applications, such as wide-band radomes, multifrequency electromagnetic shields, and satellite communications, the out-of-band performance is critical to improve selectivity. At present, the existing FSS studies involving out-of-band performance are mainly limited to single-passband FSSs [11,12,13,14]. For multi-band FSS studies, only [15] implemented a tri-band characteristic, while generating two stop-bands from 10 to 17 GHz and 40 to 50 GHz. Therefore, it remains unexplored how to generate more than three passbands while retaining good out-of-band rejection performance.

Motivated by this fact, this paper proposes a quad-passband FSS with great out-of-band rejection. Compared with that in [15], the proposed FSS realizes more passbands and has a wider stop-band range. An equivalent circuit model (ECM) is presented to demonstrate the construction principle of this design. Furthermore, it is stable for polarization and angle. The simulation and measurement results validate the performance of our designed structure.

## 2. FSS Description and Simulation Results

### 2.1. FSS Structure Design and Equivalent Circuit Model Construction

**Figure 1:**
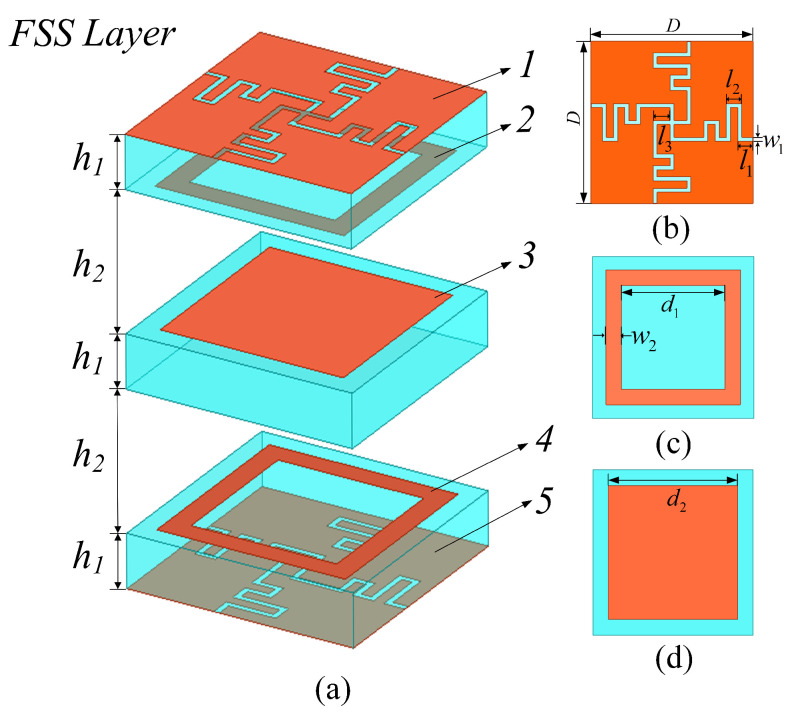
(**a**) Unit cell of the proposed FSS. (**b**) Layers 1 and 5 of the unit cell. (**c**) Layers 2 and 4 of the unit cell. (**d**) Layer 3 of the unit cell. The specific sizes are *D* = 6 mm, $l_{1}$ = 0.55 mm, $l_{2}$ = 0.57 mm, $l_{3}$ = 0.625 mm, $w_{1}$ = 0.15 mm, $w_{2}$ = 0.57 mm, $d_{1}$ = 3.86 mm, $d_{2}$ = 4.85 mm, $h_{1}$ = 1 mm, and $h_{2}$ = 2.6 mm.

Figure 1 displays the proposed FSS unit structure, consisting of multiple metal layers, dielectric substrates, and air cavities, where layer 1 and layer 5 are the same winded cross-slot layers, layer 2 and layer 4 are the same square ring layers, and layer 3 is a square patch layer. The dielectric substrates of F4BM-2 have a thickness of $h_{1}$ and a dielectric constant $\varepsilon_{r}$ of 2.65. The height of the air cavity was set to $h_{2}$.

**Figure 2:**
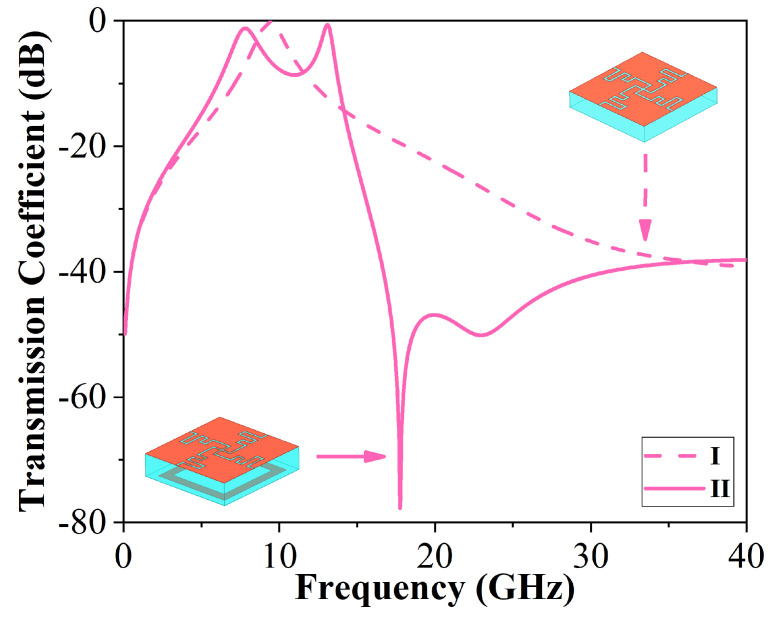
Transmission coefficients in the proposed unit evolution.

Figure 2 displays the step-by-step process of design. To illustrate the forming mechanism of the quad-band, separate and joint simulations of the winded cross-slot and square ring structure were performed in curves I and II, respectively. It can be seen in curve II that two transmission poles are produced simultaneously at two metal structure resonant frequencies. Through the simulation, combination, and improvement of the structure, it can be deduced from curves I and II that two sets of resonators connected in parallel can generate four passbands. In order to obtain the suppression of the high-frequency band range, a square patch is employed in this design that is equivalent to a capacitive.

**Figure 3:**
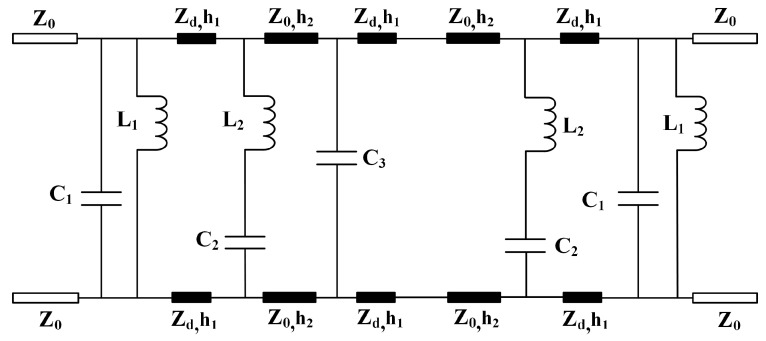
Established ECM of the designed FSS. $L_{1}$ = 0.23 nH, $L_{2}$ = 0.158 nH, $C_{1}$ = 2.591 pF, $C_{2}$ = 0.374 pF, $C_{3}$ = 0.28 pF.

To further understand the operating mechanism of the proposed FSS, the ECM was established as shown in Figure 3. The dielectric substrate with a thickness of $h_{1}$ and an air cavity a thickness of $h_{2}$ can act as a short transmission line with a characteristic impedance of $Z_{d}=Z_{0}/\sqrt{\varepsilon_{r}}=231.6\;\Omega$ and $Z_{0}=377\;\Omega$, respectively. The winded cross-slot and metallic square ring array can be equivalent to the parallel LC resonator $\left(L_{1}-C_{1}\right)$ and serial LC resonator $\left(L_{2}-C_{2}\right)$, respectively. The capacitor $C_{3}$ represents the square patch. Due to the complexity of the circuit, the coupling effects between the metal layers are not considered. By coupling among the cascaded multilayer structure, the quad-band and out-of-band suppression characteristic can be obtained.

The next step is to discuss the mapping relationship between the equivalent circuit parameters and the dimensions of the FSS. First of all, the equivalent circuit parameters are derived from the curve fitting method [16]. The dimensions of the winded cross-slot layer can be optimized by the full-wave EM simulation results and the ECM results [17]. Based on [18], the equivalent circuit parameters for the square patch array can be calculated as follows:

(1) $$C_{3}=\varepsilon_{0}\varepsilon_{eff}\frac{2D}{\pi }\ln\left(\frac{1}{\sin\frac{\pi (D-d_{2})}{4D}}\right)$$

where $\varepsilon_{0}$ is the permeability of free space and $\varepsilon_{eff}=\left(\varepsilon_{r}+1\right)/2$ is the relative permittivity of the dielectric substrate.

The evaluations of the equivalent circuit parameters for the square ring array can be obtained using the following formula [19]:

(2) $$L_{2}=\frac{X_{2}}{Z_{0}\omega }=\frac{\left(d_{1}+2\omega_{2}\right)F\left(D,2\omega_{2},\lambda \right)}{D\omega }$$

(3) $$C_{2}=\frac{B_{2}}{Y_{0}\omega }=\varepsilon_{eff}\frac{4\left(d_{1}+2\omega_{2}\right)F\left(D,D-d_{1}-2\omega_{2},\lambda \right)}{D\omega }$$

where

(4) $$F\left(D,\omega_{2},\lambda \right)=\frac{D}{\lambda }\left[\ln\left(\csc\frac{\pi \omega }{2D}\right)+G\left(D,\omega ,\lambda \right)\right]$$

(5) $$G\left(D,\omega_{2},\lambda \right)=\frac{0.5{\left(1-\beta^{2}\right)}^{2}\left[2\left(1-\frac{\beta^{2}}{4}\right)A+4\beta^{2}A^{2}\right]}{\left(1-\frac{\beta^{2}}{4}\right)+2\beta^{2}\left(1+\frac{\beta^{2}}{2}-\frac{\beta^{4}}{8}\right)A+2\beta^{6}A^{2}}$$

(6) $$A=\frac{1}{\sqrt{1-{\left(\frac{D}{\lambda }\right)}^{2}}}-1$$

(7) $$\beta =\sin\left(\frac{\pi \omega }{2D}\right).$$

The $X_{2}$ and $B_{2}$ stand for the normalized inductive reactance and the normalized capacitive reactance of the square ring array, respectively.

### 2.2. Performance Simulation and Analysis

**Figure 4:**
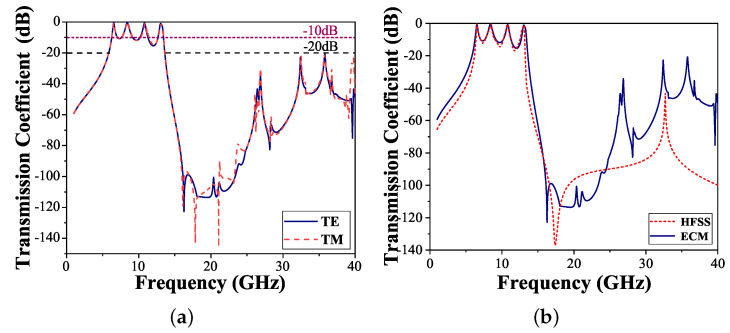
Comparison of the proposed FSS transmission coefficient. (**a**) TE and TM. (**b**) HFSS and ECM.

HFSS and ADS were used to simulate the transmission characteristics of the proposed FSS structure. Figure 4a shows the transmission coefficient curves in TE and TM modes. It can be observed that the proposed structure provides a quad-band FSS. The transmission poles are $f_{p1}$ = 6.54 GHz, $f_{p2}$ = 8.41 GHz, $f_{p3}$ = 10.80 GHz, and $f_{p4}$ = 13.06 GHz, respectively. The −3 dB bandwidths of the passbands are 6.42–6.63, 8.23–8.62, 10.75–10.95, and 12.97–13.26 GHz, respectively. The insertion losses of the four passbands are 0.77, 0.45, 0.71, and 1.11 dB, respectively. In addition, the transmission coefficient between the operating bands is below −10 dB, in the ranges of 1 to 5.5 GHz and 14 to 40 GHz, and the transmission coefficient is below −20 dB. Here, we define the frequency difference as the bandwidth of the transition band for judging the roll-off performance when the transmission coefficient drops sharply from −3 dB to −20 dB. The bandwidths of the transition band located on both sides of the passbands are 0.69 GHz and 0.57 GHz, respectively. The narrow transition bandwidths provide the FSS with a highly selective response. Figure 4a also shows that this structure has good polarization stability; the transmission coefficient curve fitting of the structure is consistent in the TE and TM modes. In comparison, the transmission coefficients obtained using the software HFSS 2019 and ADS 2019 are shown in Figure 4b. Although there are some subtle differences, it can be seen that the simulation results of HFSS and ECM are in good agreement, and the suppression effect of both is the same.

**Figure 5:**
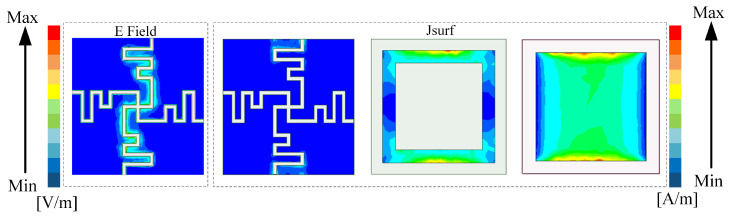
Surface current and electric field distributions at 6.54 GHz.

For further verification of the working principle of the multilayer structure, the surface current and electric field distributions at a resonant frequency were determined, and they are shown in Figure 5. Because the surface currents of each layer flow through essentially the same region at the four resonant frequencies, we provide the surface current and electric field distributions at 6.54 GHz. In the winded cross-slot region, the surface current is weak and the electric field is strong. The electric field is mainly concentrated in the bending gap, which can be modeled as a series LC resonator. It can be seen that there is almost no attenuation of the electrical intensity of the incident wave after passing through the winded cross-slot region. The surface current at the square ring region is distributed along both sides of the loop. At the square patch region, the current flows mainly predominantly throughout the area.

**Figure 6:**
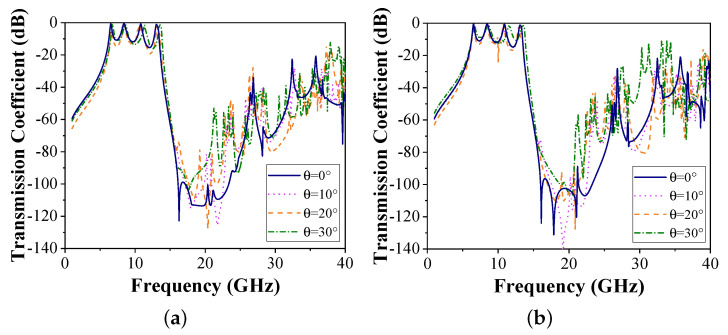
Simulated transmission coefficients under different incidence angles and polarizations. (**a**) TE polarization. (**b**) TM polarization.

The transmission coefficient curves of the proposed FSS under different incident angles for both polarizations are depicted in Figure 6. It is observed that the frequency response of the proposed FSS is basically stable for TE and TM polarization with an incidence angle up to 30°. However, as incidence angle increases, the latter two passbands tend towards high frequency. This is because the grating lobe is delayed until higher frequencies appear [1]. For TE polarization, the transmission coefficient remains below −20 dB within the 37 GHz frequency band. For TM polarization, the transmission coefficient shifts slightly upward at 30–34 GHz when the incidence angle reaches 30°. However, the proposed FSS still maintains stable transmission performance. As the incidence angle of TM polarization increases, a ripple resonance occurs between the second and third passbands. The ripple resonance has a negligible effect on the passband.

## 3. Experimental Verification

**Figure 7:**
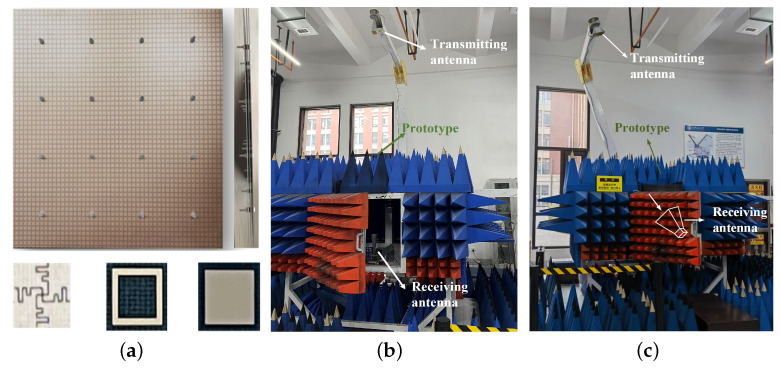
(**a**) Photograph of the fabricated FSS structure. (**b**) Photograph of free-space measurement environment. (**c**) Photograph of measurement environment under oblique incidence.

In order to further validate the feasibility of the designed FSS, a prototype with a size of 300 mm × 300 mm, consisting of 50 × 50 elements, was fabricated using three F4BM-2 boards, as presented in Figure 7a.

The thickness of the F4BM-2 boards was 1mm. The FSS layers were connected by nylon studs and nylon nuts to ensure 2.6 mm high air cavities between the three substrates. The FSS prototype was measured in a microwave anechoic chamber using the free-space measurement method. Two standard gain horn antennas acted as the transmitting and receiving antennas. As shown in Figure 7b,c, the prototype was placed in the far-field range of the transmitting antenna and directly above the receiving antenna. The vector network analyzer (VNA) was used to measure the transmission coefficient of the prototype. To cover the wide measurement frequency range, three pairs of standard horn antennas operating at 1–20 GHz, 18–26 GHz, and 26–40 GHz were used.

**Figure 8:**
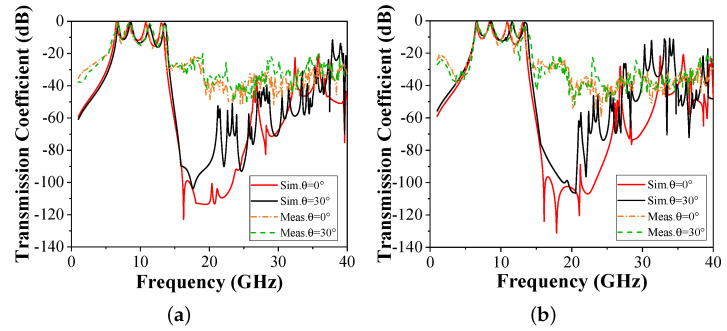
Measured transmission coefficients of the structure under oblique angles and polarizations. (**a**) TE polarization. (**b**) TM polarization.

The comparison between the measured and simulated results under oblique incidences is shown in Figure 8. As observed, the measured result is similar to the simulated result. In actual measurement, the lower transmission coefficient curve is difficult to measure. Hence, there is a difference in transmission depth between the measured results and the simulated results. The deviations in measurement results can be attributed to the fix error when the height of the air cavity is inaccurate. Nevertheless, the four passbands and out-of-band rejection characteristics can still be observed.

Subsequently, a comparison of the proposed structure with those from existing studies is presented in Table 1. It can be seen that the proposed FSS not only considers more passbands but also considers out-of-band suppression.

## 4. Conclusions

A highly selective quad-band FSS in C/X/Ku bands is proposed in this paper. ECM and full-wave simulation results indicated that the proposed FSS has a transmission response and out-of-band suppression performance. This design achieved a quad-band passband FSS with -20 dB out-of-band suppression in 1–5.5 GHz and 14–40 GHz. Additionally, the measured results are consistent with the simulated ones under an oblique angle for both TE and TM polarizations. The proposed highly selective quad-band FSS could be used for reducing the out-of-band RCS of narrowband antennas.

## Figures and Tables

**Table 1:** Comparison with existing studies.

Ref.	No. of Passbands	Element Size ($\lambda_{0}$)	−20 dB STOPBANDS (GHz)		Layers
			Lower Side	Upper Side	
[10]	4	0.14	N/A	N/A	3
[11]	1	0.07	N/A	5–40	5
[12]	1	0.21	N/A	24–30	3
[13]	1	About 0.2	N/A	2.4–10	5
[14]	1	0.33	1–9.1 (−10 dB)	11–24	3
[15]	3	0.15	10–17	40–50	3
This study	4	0.13	1–5.5	14–40	5

## Data Availability

Data are contained within the article.
